# Whole exome sequencing in dense families suggests genetic pleiotropy amongst Mendelian and complex neuropsychiatric syndromes

**DOI:** 10.1038/s41598-022-25664-7

**Published:** 2022-12-07

**Authors:** Suhas Ganesh, Alekhya Vemula, Samsiddhi Bhattacharjee, Kezia Mathew, Dhruva Ithal, Karthick Navin, Ravi Kumar Nadella, Biju Viswanath, Patrick F. Sullivan, The ADBS Consortium, Naren P. Rao, Janardhanan C. Narayanaswamy, Palanimuthu T. Sivakumar, Arun Kandasamy, Muralidharan Kesavan, Urvakhsh Meherwan Mehta, Ganesan Venkatasubramanian, John P. John, Odity Mukherjee, Ramakrishnan Kannan, Bhupesh Mehta, Thennarasu Kandavel, B. Binukumar, Jitender Saini, Deepak Jayarajan, A. Shyamsundar, Sydney Moirangthem, K. G. Vijay Kumar, Bharath Holla, Jayant Mahadevan, Jagadisha Thirthalli, Prabha S. Chandra, Bangalore N. Gangadhar, Pratima Murthy, Mitradas M. Panicker, Upinder S. Bhalla, Sumantra Chattarji, Vivek Benegal, Mathew Varghese, Janardhan Y. C. Reddy, Padinjat Raghu, Mahendra Rao, Sanjeev Jain, Meera Purushottam

**Affiliations:** 1 Central Institute of Psychiatry, Kanke, Ranchi, India; 2 Schizophrenia Neuropharmacology Research Group, Department of Psychiatry, Yale University School of Medicine, New Haven, USA; 3 Molecular Genetics Laboratory, Department of Psychiatry, National Institute of Mental Health and Neuro Sciences, Bengaluru, India; 4 National Institute of Biomedical Genomics, Kalyani, India; 5 Department of Psychiatry, Varma Hospital, Bhimavaram, India; 6 University of North Carolina at Chapel Hill, Chapel Hill, NC USA; 7 Department of Medical Epidemiology and Biostatistics at Karolinska Institutet, Stockholm, Sweden; 8 Institute for Stem Cell Biology and Regenerative Medicine (InStem), Bengaluru, India; 9 National Center for Biological Sciences (NCBS), Bengaluru, India

**Keywords:** Genetics of the nervous system, Human behaviour, Genomics, Sequencing, Medical research, Genetics, Genomics, Neurodevelopmental disorders, Sequencing

## Abstract

Whole Exome Sequencing (WES) studies provide important insights into the genetic architecture of serious mental illness (SMI). Genes that are central to the shared biology of SMIs may be identified by WES in families with multiple affected individuals with diverse SMI (F-SMI). We performed WES in 220 individuals from 75 F-SMI families and 60 unrelated controls. Within pedigree prioritization employed criteria of *rarity, functional consequence,* and *sharing* by ≥ 3 affected members. Across the sample, gene and gene-set-wide case–control association analysis was performed with Sequence Kernel Association Test (SKAT). In 14/16 families with ≥ 3 sequenced affected individuals, we identified a total of 78 rare predicted deleterious variants in 78 unique genes shared by ≥ 3 members with SMI. Twenty (25%) genes were implicated in monogenic CNS syndromes in OMIM (OMIM-CNS), a fraction that is a significant overrepresentation (Fisher’s Exact test OR = 2.47, p = 0.001). In gene-set SKAT, statistically significant association was noted for OMIM-CNS gene-set (SKAT-p = 0.005) but not the synaptic gene-set (SKAT-p = 0.17). In this WES study in F-SMI, we identify private, rare, protein altering variants in genes previously implicated in Mendelian neuropsychiatric syndromes; suggesting pleiotropic influences in neurodevelopment between complex and Mendelian syndromes.

## Introduction

Neuropsychiatric syndromes, such as schizophrenia (SCZ), bipolar disorder (BD), obsessive compulsive disorder (OCD) and substance use disorders (SUDs) (referred hereafter as serious mental illness [SMI]) often cluster in families^1,2^. Next generation sequencing (NGS) can be used to explore the genetics of complex, common disorders, using both case–control and family-based designs^3^. These include common variant genome wide association studies (GWAS) and exome wide studies that scan for rare coding variants^4,5^. Such methods have provided useful details about the contribution of rare variants to the genetic architecture of SMI, often complementing the common variant contributions identified in GWAS.

Case–control studies typically exclude relatives to minimize sampling bias and potential false positive associations. About 10% of persons affected with SMI have an affected first degree relative. The increased occurrence of potential disease relevant variants, in densely affected pedigrees, may offer clues towards genes and pathways involved in the neurobiology of SMI. Many studies employing whole exome sequencing (WES) in psychiatry have analyzed families with multiple affected members (reviewed in^6^). These studies have generally focused on pedigrees with a cluster of individuals affected with a specific SMI such as BD or SCZ. Family-based studies in SCZ^7–9^, BD^10–13^ and OCD^14^ have identified multiple rare de novo and loss of function variations relevant to the biology of each syndrome. Similarly, case–control association studies in SCZ^4,5^ and BD^15,16^ have also identified the contribution of rare variants of large effect, advancing the current understanding of genetic architecture of these syndromes. An overview of recent findings from family based and case–control sequencing studies in SMI is presented in the Supplement S1.

In summary, we note a trend towards a higher burden of rare, protein altering variations, across cases with different SMI syndromes, when compared to controls^17,18^. These variants tend to be overrepresented in genes that are integral to neurodevelopment and synaptic function, and these ontologies are often identified across SMI syndromes^19–21^. Some genes with a higher burden of rare variants, identified in the family studies, have also been implicated in other severe neuropsychiatric syndromes, with features of anomalies in neurodevelopment and neurodegeneration^22,23^. There seems to be considerable overlap of rare variants that contribute to the risks of SMI, across syndromes. This convergence has also been observed for common variants^24^, as well as across genes, gene networks^25,26^, molecular pathways^27^, and brain imaging endophenotypes^28^. A recent analyses that integrated GWAS findings across 11 major psychiatric syndromes, suggests a shared genetic architecture across syndromes at bio-behavioural, functional genomic and molecular genetic levels^29^. A co-aggregation of SMI syndromes, and overlapping symptom dimensions, are often seen within a family^1,2^. Rare variants, identified in families with multiple ill members, may thus explain a proportion of the risk in the population. They are obviously of great heuristic value, to explore the pathobiology of the disease, and correlates of clinical features.

In this study, we examined the occurrence of rare, deleterious variants in individuals from families that had multiple affected members (as identified in Accelerator Program for Discovery in Brain Disorders using Stem Cells (ADBS))^30^. Within pedigree segregation, as well as cross-sample case–control association tests, were used to prioritize risk variants. We examined the functional and clinical significance of the prioritized genes and variants, including evolutionary conservation, mutation intolerance, brain expression, protein function and disease relevance. Specifically, we examined if the genes carrying the prioritized variants are overrepresented in Mendelian neuropsychiatric syndromes, and synaptic genes (as attempted for SCZ in the Xhosa population^4^). In families segregating a variant in a gene linked to a syndrome, we also reviewed the clinical profile of affected individuals for symptoms and signs of the particular Mendelian syndrome.

## Results

### Sample

We sequenced 280 (131 females) individuals, including 220 from 75 families (F-SMI), and 60 unrelated-controls. Of the F-SMI samples, 160 were cases diagnosed with a SMI: SCZ (n = 63), BD (n = 80), OCD (n = 7), SUD (n = 7), complex SMI (n = 3) along with 60 family-controls without a lifetime diagnosis of mental illness. The demographic profile of the sample with age and sex distribution and illness profile of the affected sample is provided (Table 1). Fifty-one (68%) of the 75 families had cases with diagnosis across ≥ 2 of the 4 illness categories noted above.

**Table 1:** Demographic and clinical profile of the sample.

1a. Demographic profile of cases, family controls and unrelated controls					
Groups	Cases	Family controls	Unrelated controls	F/Chi	p
Sample size	160	60	60		
Age—mean (SD)	39 (13.5)	43.8 (15.3)	45.6(18.2)	5.13	0.006
Sex male/female	92/68	28/32	29/31	2.79	0.25

1b. Clinical profile by diagnosis					
Diagnosis	Sample size	Age Mean (SD)	Sex (male/female)	AAO Mean (SD)	DOI Mean (SD)
Schizophrenia	63	37.1 (12.9)	35/28	23.1 (7.5)	12.4 (9.8)
Bipolar disorder	80	39.9 (13.5)	45/35	21.9 (7.5)	19.9 (11.3)
OCD	7	33.7 (12.1)	2/5	24.4 (7.7)	8.4 (7.3)
Addiction	7	42.9 (13.9)	7/0	33.8 (14.4)	9 (7.9)
Mixed SMI	3	46 (22.9)	3/0	41.6 (14.2)	8 (8.2)

*AAO* age at onset, *DOI* duration of illness, *SD* standard deviation.

Within the F-SMI familial sample, the median number of samples from cases per family were 2 (range: 1–6) and family-controls was 1 (range:1–7). A subset of 16 multi-sample F-SMI (≥ 3 exome samples) was selected for analysis of within pedigree segregation of putatively deleterious variants.

### Variant profile

**Figure 1:**
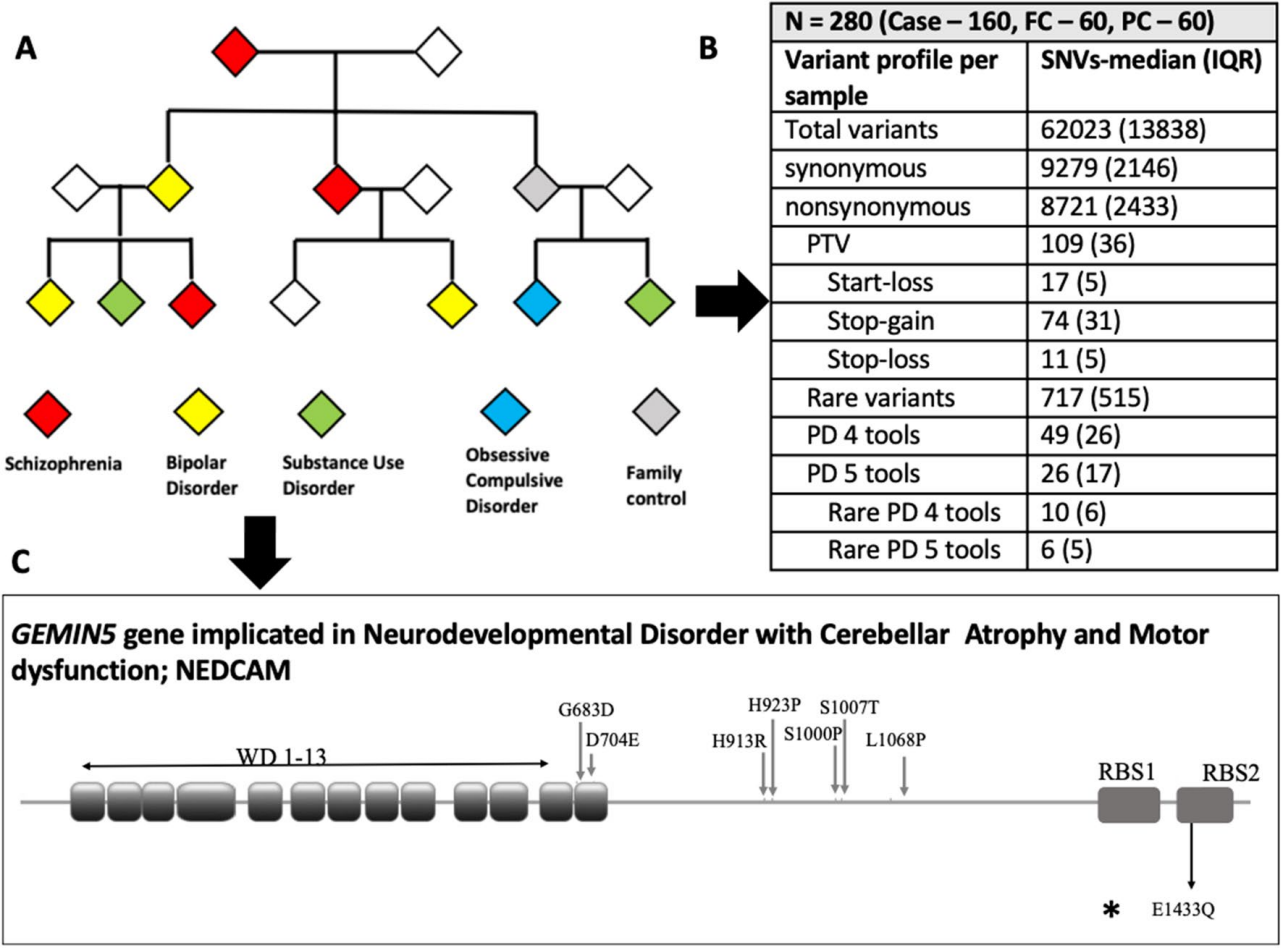
Conceptual overview—exome sequencing was done for individuals from families with multiple members with serious mental illness (SMI). The table shows the meaningful (significant) variants detected. Details of one representative gene (GEMIN5) are shown. The seven variants marked above have been implicated in Neurodevelopmental Disorder with Cerebellar Atrophy and Motor dysfunction: NEDCAM earlier. The variant marked below rs544452250 (5-154268943-C-G; E1433Q) was predicted to be deleterious, and was shared across three individuals with schizophrenia, BPAD and Substance use disorder, in one family.

Variant calling the exomes of 280 samples resulted in identification of 793,818 unique variants. Among these, the median (interquartile range) number of synonymous and non-synonymous SNVs per sample were 9279 (2146) and 8721 (2433) respectively. The median (interquartile range) count of rare variants (minor allele frequency [MAF] < 0.1%) per sample was 717 (515). A breakdown of the variant profile in the sample is provided in Fig. 1. A total of 3556 unique single nucleotide polymorphisms and small insertion deletions were defined as rare putatively deleterious (RPD) variants based on the criteria of rarity (-MAF < 0.1%) and deleteriousness (loss of function or missense predicted deleterious) as further enumerated in the methods.

### Within pedigree segregation of private variants

Among 14 of the 16 multi-sample high-density families, we identified a total of 78 RPD variants in 78 different genes that were shared by ≥ 3 affected members within a family and were absent in the 60 unrelated-controls, defined as shared-broad RPD (sb-RPD) (Supplementary Table 1). 77 variants were private to one among the 14 pedigrees. Only the variant at the position chr5:154268943 in *GEMIN5* gene was shared by 3/6 and 3/3 cases from families D002 and D012 respectively (Fig. 1, Table 2).

**Table 2:** Variants segregating within families with SMI in genes implicated in Mendelian syndromes.

chr:position	Reference>alternate	Gene^a^	Family number	Burden-case	Gene description	OMIM syndrome
chr18:12337480	G>C	***AFG3L2***	D002	3	AFG3 like matrix AAA peptidase subunit 2	Spinocerebellar Ataxia 28; SCA28
chr17:3384920	A>G	***ASPA***	D002	3	Aspartoacylase	Canavan-Van Bogaert-Bertrand disease—spongy degeneration of central nervous system
chr9:141016353	C>T	***CACNA1B***	D002	4	Calcium voltage-gated channel subunit alpha1 B	Neurodevelopmental disorder with seizures and nonepileptic hyperkinetic movements
chr11:64953762	G>A	***CAPN1***	D002	3	Calpain 1	Spastic paraplegia 76, autosomal recessive
chr20:10625568	C>G	***JAG1***	D002	5	Jagged canonical Notch ligand 1	Alagille syndrome 1
chr1:10035801	C>T	***NMNAT1***	D002	3	Nicotinamide nucleotide adenylyltransferase 1	Spondyloepiphyseal dysplasia, sensorineural hearing loss, impaired intellectual development, and leber congenital amaurosis; shilca
chr5:154268943	C>G	***GEMIN5***	D002	3	Gem nuclear organelle associated protein 5	Neurodevelopmental disorder with cerebellar atrophy and motor dysfunction; NEDCAM
chr5:154,268,943	C>G	***GEMIN5***	D012	3	Gem nuclear organelle associated protein 5	Neurodevelopmental disorder with cerebellar atrophy and motor dysfunction; NEDCAM
chr21:47860904–47860904	–>G	***PCNT***	D003	6	Pericentrin	Microcephalic osteodysplastic primordial dwarfism, type II
chr12:120241184	G>A	***CIT***	D004	3	Citron rho-interacting serine/threonine kinase	Microcephaly 17
chr10:70225532	G>A	***DNA2***	D006	4	DNA replication helicase/nuclease 2	Seckel syndrome 8
chr2:166032822	G>A	***SCN3A***	D006	4	Sodium voltage-gated channel alpha subunit 3	Epilepsy familial focal with variable foci 4, developmental and epipeltic encephalopathy 62
chr12:23998998	C>T	***SOX5***	D006	4	SRY-box transcription factor 5	Lamb Shaffer syndrome
chr2:69409769	G>T	***ANTXR1***	D007	3	ANTXR cell adhesion molecule 1	Growth retardation, alopecia, pseudoanodontia, and optic atrophy
chr17:40715328	C>T	***COASY***	D007	3	Coenzyme A synthase	Neurodegeneration with brain iron accumulation 6, pontocerebellar hypoplasia, type 12
chr3:143567048	C>T	***SLC9A9***	D007	3	Solute carrier family 9 member A9	Autism susceptibility AUTS 16
chr6:31827960	G>A	***NEU1***	D008	3	Neuraminidase 1	Neuraminidase deficiency
chr11:66029625	T>C	***KLC2***	D009	3	Kinesin light chain 2	Spastic paraplegia, optic atrophy, and neuropathy; SPOAN
chr5:178581083	C>T	***ADAMTS2***	D010	3	ADAM metallopeptidase with thrombospondin type 1 motif 2	Ehlers-Danlos syndrome, dermatosparaxis type
chr6:43018723	C>T	***CUL7***	D013	3	Cullin 7	Three M syndrome 1
chr19:50826985	C>T	***KCNC3***	D013	3	Potassium voltage-gated channel subfamily C member 3	Spinocerebellar ataxia 13; SCA13

^a^The underlined genes are those that harbored shared-stringent variants defined by stringent prioritization approach.

Of the 78 prioritized sb-RPD variants, 46 variants had MAF < 0.01% and 36 of these were absent or noted in no more than one unaffected family control and were defined as shared-stringent-RPD (ss-RPD) (Supplementary Table 1a).

In the set of 59 pedigrees with ≤ 2 case samples, 15 of the same 78 genes harboring sb-RPD were noted to carry RPD variants that were absent in the 60 unrelated-controls (Supplementary Table 2). Additionally, at the variant level, 4 of the 78 RPD variants prioritized from the set of 14 families, were also noted in this latter set of 59 families (Supplementary Table 3).

### Overrepresentation analysis

Twenty of these 78 genes (25%) in the sb-RPD set are implicated in monogenic neurodevelopmental syndromes, with both dominant and recessive modes of inheritance in the Online Mendelian Inheritance in Man (OMIM) database. We performed an overrepresentation analysis of our gene-list on a list of 2450 genes having a Central Nervous System (CNS) phenotype annotation in OMIM clinical synopsis (OMIM-CNS) among 20,203 protein coding genes in the human genome (Supplementary Table 4). The prioritized gene list from multi-sample F-SMI was significantly enriched for genes implicated in monogenic CNS syndromes (Fisher’s Exact test OR = 2.51, 95%CI = 1.43–4.25, p = 0.001). These gene-phenotype relationships along with the genomic coordinates and pathogenicity prediction of the identified variants in this sample are described in Table 2.

The effect size of the overrepresentation was much larger in the gene harboring ss-RPD subset, defined with stringent prioritization criteria as 13 out of 36 prioritized genes (37%) were noted in OMIM-CNS gene-set (Fisher’s Exact test OR = 4.11, 95%CI = 1.91–8.48, p = 0.0002). These genes are marked within Table 2.

To check if the observed overrepresentation was specific to the CNS, we derived 19 additional gene-lists that encompassed genes implicated in OMIM syndromes affecting other organ systems (e.g. ‘cs_head_and_neck_head’, ‘cs_cardiovascular’ ‘cs_respiratory’ etc.). Among the 20 OMIM derived gene-lists (CNS+ above 19), for the 78 sb-RPD gene set, statistically significant overrepresentation at p < 0.0025 (0.05/20) was noted for gene lists annotated with clinical synopsis terms ‘central nervous system’ (p_cor_ = 0.026), ‘head and neck’ (p_cor_ = 0.037), and a nominally significant overrepresentation for ‘peripheral nervous system (0.057) (Supplement S2 and S3 and Supplementary Table 4). Among the 36 genes in ss-RPD gene-set, across 20 OMIM clinical synopsis term gene lists, only OMIM-CNS list derived from clinical synopsis term ‘central nervous system’ (p_cor_ = 0.011) showed statistically significant overrepresentation. These results suggest that the prioritized gene lists harboring sb-RPD and ss-RPD variants were specifically overrepresented in clinical conditions involving these systems.

We performed an overrepresentation analysis in a set of genes (n = 516) carrying RPDs in the 60 unrelated controls that MAF < 0.01% in gnomAD South Asian and global samples similar to the threshold used in defining ss-RPD subset. While we noted a statistically significant overrepresentation (OR = 1.77, 95%CI = 1.41 to 2.22), the magnitude of effect was significantly lower when compared to the effect size noted for the ss-RPD gene-set (OR = 4.11, 95%CI = 1.91 to 8.48) consisting of 36 genes prioritized in within-pedigree analysis (z = 2.03, one tailed p-value = 0.01). Furthermore, the observed overrepresentation for control gene-set harboring RPDs was nonspecific as significant effects were noted for 13 out of 20 OMIM clinical synopsis genes-lists.

We also compared the effect size of the overrepresentation analysis statistic between genes harboring ‘segregating’ and ‘non-segregating’ RPDs among the cases within affected pedigrees. The magnitude of effect for overrepresentation was lower in the ‘non-segregating’ RPD gene set compared to segregating gene set (Supplementary S3, Supplementary Fig. 1).

Unlike the OMIM-CNS gene-list, we did not note an overrepresentation of the synaptic gene-list (1233 genes) from SynGO database^31^ for the set of 78 genes with sb-RPD variants segregating within pedigrees (Fisher’s Exact test OR = 1.5, 95%CI = 0.58–3.26, p = 0.34) or the 36 genes with ss-RPD variants (Fisher’s Exact test OR = 1.4, 95%CI = 0.28–4.62, p = 0.47).

### Functional significance of prioritized genes

These 78 prioritized genes with sb-RPD variants in the within-pedigree analysis were significantly more conserved, in comparison with the background list of 20,125 remaining protein coding genes (mean (SD) conservation score (Zoonomia^32^) 0.63 (0.13) vs 0.59 (0.18), p = 0.014). Among these genes, 20 OMIM-CNS genes were significantly more mutation intolerant as compared to the other 59 genes, as suggested by lower LOEUF score^33^ ((mean (SD) − 0.6 (0.32) vs 0.99 (0.48), p = 0.001)) and a higher pLI score^34^ ((mean (SD) − 0.41 (0.49) vs 0.16 (0.36), p = 0.05)). The mean expression of these 20 genes was also significantly higher compared to the other 59 genes in the brain cortex (mean (SD) − 15.9 (15.3) vs 5.2 (7.5), p = 0.006); and rest of the brain (mean (SD) − 16.9 (15.2) vs 6.02 (8.4), p = 0.005).

### Cross-pedigree association analysis using Sequence Kernel Association Test (SKAT)

We performed a geneset-level association analysis to examine genes with higher burden of RPD variants compared to controls. The analysis adjusted for kinship structure and population structure inferred by Principal Component Analysis. This analysis included two candidate gene-sets, viz. synaptic gene-set (1233 genes) from SynGO database^31^ and the OMIM-CNS gene-set (2450 genes) to examine the difference in the association of RPD variant burden in each of these gene-sets among cases and controls using SKAT (by treating these gene sets as a pooled SNP-set). After correcting for kinship and population structure we noted a statistically significant association for OMIM-CNS gene-set (p_bonferroni_ = 0.005) but did not note a statistically significant association for the synaptic gene-set (p_bonferroni_ = 0.17) (Table 3). These results remained robust in a sensitivity analysis that involved association analysis in ss-RPD variants with MAF < 0.01% with a significant association for OMIM-CNS gene-set (p_bonferroni_ < 1e−7) but not for the synaptic gene-set (p_bonferroni_ = 0.24). In a preliminary gene-level SKAT analysis, we noted genome wide significance (p < 2.4e−6) for a single gene *POTEE* and nominally significant associations (p < 0.05) for 9 additional genes with a higher variant burden among cases (Supplementary Table 5).

**Table 3:** Gene-set level cross-pedigree association analysis.

Gene-set association analysis				
Gene-set	Variant count	Case variant count	UC variant count	SKAT p value
OMIM CNS genes	568	646	245	0.005
Synaptic genes	186	321	76	0.17

## Discussion

Employing WES in families with multiple members detected to have SMIs, we identify segregation of rare deleterious variants in several genes. A significant proportion of these genes are implicated in neuropsychiatric syndromes with Mendelian inheritance, such as *(GEMIN5, COASY, CACNA1B* etc., see Table 2). Majority (78/79) of the prioritized variants were noted to be private to fourteen multi-sample pedigrees. In four of these families, review of health records revealed evidence of clinical features that overlapped with the primary OMIM syndrome. The 79 genes prioritized by pedigree analysis tended to be more conserved, and more intolerant of variation, when compared to the remaining set of 20,124 genes. In addition, in the cross-pedigree association analysis, cases harbored an increased burden of rare deleterious variants in genes involved in the structural and functional integrity of synapses, as compared to unrelated controls.

The overrepresentation of genes in within-pedigree prioritization was largely specific to disorders of ‘central nervous system’ (CNS), as genes with a clinical synopsis annotation to this category showed the strongest overrepresentation. Among 19 additional clinical synopsis categories, significant overrepresentation was noted for ‘head and neck’ term. This overlap with ‘head and neck’ may be explained by tightly interlinked developmental and molecular processes that regulate cranio-facial and brain development^35^. While there was evidence for increased burden of RPDs in genes relevant to CNS, we did not find a system-specific enrichment for genes harboring RPDs among 60 unrelated controls or the genes harboring non-segregating RPDs.

Among the 20 variants in genes implicated in CNS syndromes, three variants had been previously reported in the ClinVar database in the context of the primary Mendelian syndrome (Table 2). Of these, the variants in *ADAMTS2* and *JAG1* were noted to be of ‘uncertain significance’ and *AFG3L2* variant was recorded as ‘likely benign’^36^. The remaining 17 variants have not been previously reported in the ClinVar database, in the context of a primary Mendelian syndrome. Interestingly, a duplication at the same site where we find a G allele insertion (Chr21: 47860904) in the *PCNT* gene (implicated in Microcephalic osteodysplastic primordial dwarfism type II), has been identified as a pathogenic variant in ClinVar, suggesting the significance of this site in protein function.

Pleiotropic influence for de novo variants and genes between SCZ, BD, autism and neurodevelopmental syndromes has been reported in large trio-based studies^7,18,23^. Our analysis also suggests genetic pleiotropy between Mendelian monogenic syndromes, and complex SMI syndromes. Ten families with variants in genes implicated in Mendelian neuropsychiatric syndrome did not have the ‘classical’ features of the primary syndrome. However, in four of these families we noted partial overlaps in clinical features, in some of the individuals with SMI (Supplementary Table 6). This may suggest incomplete penetrance and expression of the primary syndrome, and possible pleiotropic expression of a psychiatric syndrome for identified gene variants.

To examine if the observed pleiotropy between monogenic and complex SMI was specific to the current sample structure, population, or variant/gene prioritization approach we curated a list of 142 genes identified in 10 recent NGS studies in SCZ, BD, OCD and AD in the literature (Supplementary Table 7). Here too with 37 of 142 (26.1%) genes implicated in a Mendelian syndrome, we noted a statistically significant overrepresentation (Fisher’s Exact test OR = 2.29, p < 0.0001). Despite heterogeneity across these 10 studies with respect to the psychiatric syndrome, sample selection and variant prioritization methods, consistent evidence of overlap of risk between Mendelian and complex disease, at the level of aggregate list of genes harboring rare variants is noted.

Thus, while certain mutations in Mendelian disease genes result in early onset severe neurodevelopmental phenotype, other variants in the same genes may result in more subtle anatomical and functional consequences, that perhaps predispose to late-onset neuropsychiatric disorders^37^. These phenotype level effects of a given mutation, in a given gene, may further be moderated by background genetic effects^38,39^ and the role of the gene in neurodevelopment, and plasticity, over time^40^. Similar findings have been recently reported in a whole exome sequencing study of severe, extremely treatment resistant schizophrenia where authors note enrichment of genes with missense and loss of function variants, increased burden of Mendelian disease genes and specifically the loss of function genes to those syndromes with an annotation of behavioral phenotype^41^. Lastly, behavioral symptoms are frequently encountered with many Mendelian neurodevelopmental syndromes, and familial SMI could represent a cumulative consequence of multiple rare variants in more than one gene^42^.

Consistent with the enrichment of variants prioritized by within pedigree prioritization to OMIM-CNS gene-set, we noted a higher burden of RPD variants in the OMIM-CNS genes in across pedigree SKAT association analysis. However, a similar increased burden was not noted in the set of genes related to synaptic function. Both family-based and population-based genetic studies probing common and rare variants across a spectrum of SMI syndromes have implicated multiple synaptic genes with consequences on the structure and function of synapse^18,43–45^. As in the case–control WES study in South African Xhosa^4^, and using the same target list of genes, we observed rare predicted-deleterious variants in 104 genes related to synaptic structure and function. Of these, variants in genes represented by the GO component ‘integral component of presynaptic membrane’ (17/104) and ‘integral component of postsynaptic membrane’ (16/104), contributed the greatest proportion. Most (95%) of the rare-predicted deleterious variants in synaptic genes were private to a pedigree, or an individual, with only 6 genes having variants across two or more pedigrees (Supplementary Table 8).

In the gene-wise association test using SKAT in the cross-pedigree analysis (Supplementary Table 5), the signal noted at *POTEE* gene passed the threshold for genome-wide significance (p < 2.4E−6). Nominally significant associations were noted for nine additional genes (Supplementary Table 5). Variation in *POTEE* has not been previously reported in the context of neuropsychiatric syndromes, but differential expression of the gene product has been reported in substantia nigra in the neuroimmune syndrome such as Multiple Sclerosis^46^. *CTBP2* gene which codes for C-terminal binding protein 2, an isoform of which is a major component of synaptic ribbons, also plays a critical role in regulating cell migration during neocortical development^47^. Genome wide significant loci for multiple brain cortical morphometric measurements such as sulcal depth, cortical thickness and cortical surface area have been mapped to *ADAMTS20* gene in previous studies^48,49^. The *SLC9B1* gene is within the cis regulatory region of a genome wide significant association locus in a recently published GWAS of schizophrenia^50,51^. The *ABCD1* gene on chromosome X encodes peroxisomal membrane protein called adrenoleukodystrophy protein involved in the transport of very long chain fatty acids. More than 650 mutations in this gene have been implicated in adrenoleukodystrophy syndrome, with highly variable clinical manifestations that often include cognitive and neuropsychiatric symptoms^52^. *SYNJ2* gene has highest relative expression in human brain and encodes inositol polyphosphate phosphatase and is involved in neurotransmitter recycling. Variations in this gene have not thus far been implicated in neuropsychiatric syndromes but chromatin modification in this gene has been identified in the context of SCZ^53^. In summary, among the nominally significant gene wise associations with a higher variant burden among cases identified in this study, some had evidence in the literature with relevance to SMI and neurodevelopment syndromes but need further examination in larger samples. Given the small number of case pedigrees and control samples examined in the current study, these results warrant confirmation in future studies with larger samples from the relevant population.

Some limitations are to be considered while interpreting the results of this study. While each F-SMI pedigree had multiple members affected with SMI, we could sample 3 or more affected individuals in only 16 such pedigrees. Hence the within-pedigree segregation analysis could only be performed in this subsample, reducing the power of this analysis to detect additional disease relevant variants. Augmenting sampling in remaining pedigrees may yield larger number of SMI relevant signals. A non-uniform structure of relatedness among sequenced samples within the pedigrees and absence of a sequenced unaffected family-control in 4 of the 16 pedigrees precluded filtering variants that segregated among unaffected members in every pedigree. We were also unable to perform family-based association testing in the present analysis. However, measurement of quantitative disease relevant endophenotypes within the same pedigrees will allow us to determine the impact of RPD variants on traits within unaffected members.

We noted an overrepresentation of OMIM-CNS genes but not the synaptic genes both in the within pedigree prioritization and cross-pedigree association analysis. These results highlight the consistency in the direction of results within the study but stand in contrast to previous studies that have reported an increased burden of rare functional variants in synaptic genes. These results lead us to speculate that in the context of genetic risk for complex neuropsychiatric syndromes in familial context, two-fold burden of effects from neurodevelopmental and synaptic function genes may contribute to final disease expression. Future family studies that sequence a larger number of affected and unaffected individuals within pedigrees and in addition characterize a spectrum of phenotype effects across lifespan may yield more robust signals of overlaps between monogenic neurodevelopmental syndromes and complex SMI.

Alignment and variant calling were performed with hg19, an earlier version of reference sequence for the human genome. A recent study has demonstrated that 0.9% of exome targets and up to 206 genes may fall in regions that are susceptible to discrepancies in the reference assemblies between hg19 and hg38^54^. We verified the prioritized genes in within-family segregation and cross-pedigree association analysis and observed that none of these overlapped with the discrepant genes or regions^54^. While our sample was adequately powered to confirm cross-pedigree association in pre-identified gene sets, the novel gene-wise associations identified in this analysis are would need confirmation in larger, diverse samples.

## Conclusion

In this F-SMI WES study, we identify several private, rare, protein altering variants that segregate among the cases within a pedigree. We find suggestive evidence for overrepresentation of genes harboring these variants among genes implicated in monogenic forms of Mendelian neuropsychiatric syndromes, highlighting pleotropic influences in neurodevelopment and functioning. We also note a greater frequency of variants in genes involved in structural and functional integrity of synapses in cases compared to controls. The study demonstrates the usefulness of NGS approaches in F-SMI to identify disease relevant variants. Future studies, involving a larger number of families, with multiple affected members, and across diverse populations, may help us explore the contribution of rare coding variants to F-SMI. Validation of the functional impact of identified variants using cell models, combined with the application of in silico approaches to model protein structure alterations, and interactions, may help understand the convergent and divergent developmental mechanisms that underlie rare variants and risk of complex SMI.

## Methods

### Ethics statement

The study protocol was approved by the Institutional Ethics Committee of National Institute of Mental Health and Neurosciences, Bengaluru, India, and the study procedures conformed to the provisions of the Declaration of Helsinki. All participants provided written informed consent.

### Sample

The families with multiple members with SMI (F-SMI) were identified as part of ADBS. This program is aimed at characterization of clinical and neurobiological phenotypes for neuropsychiatric syndromes and identification of genetic and molecular correlates of disease using cell models^2,30,55^. The diagnosis of SMI was established by independent clinical evaluation by two psychiatrists based on ICD-10 criteria^56^. Diagnosis and current and lifetime comorbidity were further evaluated and confirmed with Mini International Neuropsychiatric Inventory 5.0.0^57^. The clinical status of the members belonging to the first two generations of all recruited participants was confirmed using Family Interview for Genetic Studies and pedigree charting^58^.

Cases were individuals with a diagnosis of SMI and family controls were unaffected individuals from the same families without a lifetime diagnosis of SMI or related syndromes. In addition, we identified unaffected unrelated individuals without family history of SMI, as ‘unrelated-controls’ from the population. Families with WES data from ≥ 3 cases were identified as ‘multi-sample F-SMI’ and selected for analysis of within-family segregation of variants as described below.

### Sequencing, alignment, variant calling and quality assessment

The protocols for sequencing, alignment and variant calling including the quality controls (QC) have been previously published^22^ (Supplement S5). In brief, Illumina Nextera exome enrichment kits targeting 62.08 Mb of human genome were used for library preparation and the Illumina Hiseq platform was used for 100 base paired-end sequencing. Raw-read QC was performed with FastQC.0.10.1 and low-quality reads (< Q20) were excluded. Alignment to human genome build hg19/GRCH37 was performed using BWA (v-0.5.9). Realignment was performed with 1000G Phase1 INDELs using GATK (v-3.6) for removal of PCR duplicates and alignment artifacts. Single Nucleotide Polymorphisms (SNPs) and short insertion deletions (INDEL) variants were called with standard parameters (min coverage = 8, MAF ≥ 0.25 and P ≤ 0.001) using Varscan2 to generate sample-wise VCFs.

### Annotation

VCFs were annotated with ANNOVAR^59^ tool for gene, region and filter based annotation options. Variant frequencies were obtained from the gnomAD—South Asian subset (N = 15,308). For in silico prediction of deleteriousness of coding variants, five functional annotations (*SIFT*^60^, *LRT*^61^, *MutationTaster*^62^, *MutationAssessor*^63^ and *MetaSVM*^64^) were used.

### Variant prioritization

Variants were prioritized based on rarity (minor allele frequency ≤ 0.001 in gnomAD SAS and gnomAD global samples) and a ‘predicted-deleterious’ functional consequence. Predicted-deleteriousness of exonic Single nucleotide variants (SNVs) were defined as protein truncating variants (stop-gain, stop-loss or start loss, canonical splice sites) or missense variants predicted to have deleterious consequence in ≥ 4 of the 5 functional prediction annotation tools. Protein truncating or out-of-frame small insertion-deletions (indels) were similarly prioritized. All subsequent analysis involved the prioritized rare predicted-deleterious (RPD) variants. Minor allele frequencies of the prioritized variants were examined in remaining populations in gnomAD database to exclude rare variants specific to South Asian sample.

### Analysis approach

To identify the RPDs that are putatively relevant to SMI syndromes we adopted two independent analytical approaches; a within-family prioritization of variants segregating with SMI and a cross-pedigree case–control association analysis.

#### Within-family variant selection

To identify high penetrance rare variants and to reduce potential false discoveries we defined an a priori criteria of sharing among ≥ 3 related cases with SMI within a pedigree, similar to an approach also used in a previous publication (12). To account for non-uniform relatedness structure between sampled cases across different pedigrees and the absence of an unaffected family control in 4 of the 16 multi-sample F-SMI (Supplement 6), we defined a broader set of shared RPD based on the criteria that were shared by ≥ 3 cases and were absent in unrelated technical controls from population and defined these as shared-broad RPD (sb-RPD). We further prioritized a second sub-set of shared RPDs with more stringent selection criteria and defined as shared-stringent RPD (ss-RPD). In addition to being shared by ≥ 3 cases within a pedigree, for these variants we chose a stringent MAF cut-off ≤ 0.0001 in gnomAD SAS and gnomAD global samples. Furthermore, these were either absent or were noted in not more than one in unaffected family control to allow for incomplete penetrance.

The resulting lists of shared-broad RPD (sb-RPD) and shared-stringent RPD (ss-RPD) variants and the genes harboring these variants were queried with RPDs noted in the cases in previously excluded families (< 3 samples from cases/family) to examine potential variant and gene level overlaps in SMI associated genes.

#### Case control association analysis

We performed gene-level and gene-set case–control association analysis between the cases and unrelated-controls, after accounting for the kinship and population sub-structure within the sample using Sequence Kernel Association Test (SKAT)^65^ as implemented in the R package SKAT^66^. A principal component analysis (PCA) was performed using common SNPs within the sample excluding uninformative variants to infer population sub-structure. The SKAT_null_emmax() function was used to approximately adjust for the overall genomic correlation among the individuals by fitting a null model for the binary case/control status incorporating the kinship matrix, first three principal components and three broad clusters observed in the PCA plot (Supplement S4). After covariate adjustment, the residuals obtained were permuted to obtain resampled residuals using the SKAT_Null_Model() function. Finally, association of RPD variants with case/control status was examined using SKAT() function. Up to 10^6^ permutations were used per variant to obtain accurate p values in the critical range (p > e−5). For smaller p-values, the asymptotic SKAT p-values (by “davies” method” were retained). A p value threshold of 2.4e−6 in the permuted p value was set to infer genome wide significance for gene-level analysis after Bonferroni correction for 20,203 protein coding genes. Gene-set wide analysis p values were adjusted for Bonferroni correction for the two gene-sets tested.

### Clinical and functional significance of the prioritized genes

The set of genes from within- family prioritization was examined for enrichment in genes implicated in monogenic neurodevelopmental syndromes in Online Mendelian Inheritance in Man (OMIM) database (OMIM-CNS genes) (Supplement S2) using the two tailed Fisher’s Exact test. The specificity of this enrichment to the CNS was verified by examining enrichment for 19 non-CNS gene lists among additional OMIM clinical synopsis categories (Supplement S2) and by repeating this analysis in genes harboring RPD variants in unrelated controls. In families where RPD variants in OMIM genes for particular monogenic CNS syndromes were noted, we examined the medical records for any overlapping OMIM-like clinical features. We further examined if the prioritized genes were overrepresented for synaptic genes derived from SynGO database^31^.

We examined the mutational constraint, tissue expression and evolutionary conservation using publicly available datasets. Mutational constraint was assessed by gnomAD LOEUF^33^ and ExAC pLI scores^34^. Brain and cortical expression were examined using the GTEx v8 with mean expression values per gene. Conservation scores derived from 240 species alignment were adopted from the Zoonomia consortium (Zoonomia fraction of CDS phyloP ≥ 2.270 (fdr 0.05))^32^. Comparisons between prioritized and the background list were performed using the Welch’s t-test.

## Supplementary Information

Supplementary Information 1.

Supplementary Information 2.

Supplementary Information 3.

## Data Availability

The datasets generated and/or analysed during the current study are available from the ADBS bio repository https://www.ncbs.res.in/adbs/bio-repository on registration and reasonable request.
