# Supplementary material for: Whole exome sequencing in dense families suggests genetic pleiotropy amongst Mendelian and complex neuropsychiatric syndromes
Source: Sci Rep. 2022 Dec 7;12:21128. doi: 10.1038/s41598-022-25664-7 (PMC9729597; doi:10.1038/s41598-022-25664-7)

D001

D001.1	BPAD
D001.2	BPAD
D001.3	BPAD
D001.4	BPAD
D001.5	BPAD
D001.6	Familial_control
D001.7	Familial_control

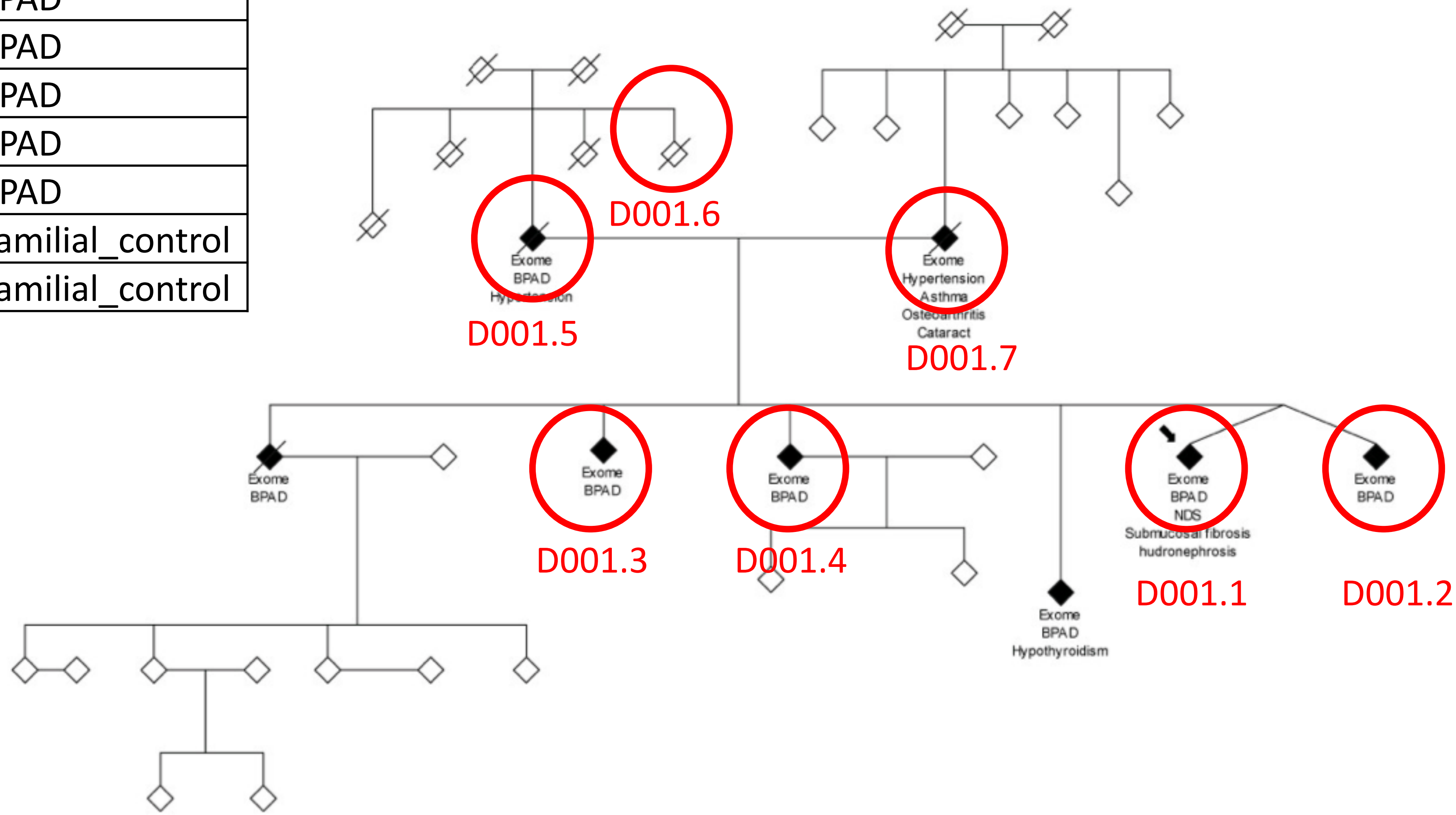

D002

D002.1	BPAD
D002.2	BPAD
D002.3	BPAD
D002.4	Depression
D002.5	Familial_control
D002.6	Familial_control
D002.7	Familial_control
D002.8	Familial_control
D002.9	Familial_control
D002.10	Familial_control
D002.11	Familial_control
D002.12	Schizophrenia
D002.13	Schizophrenia

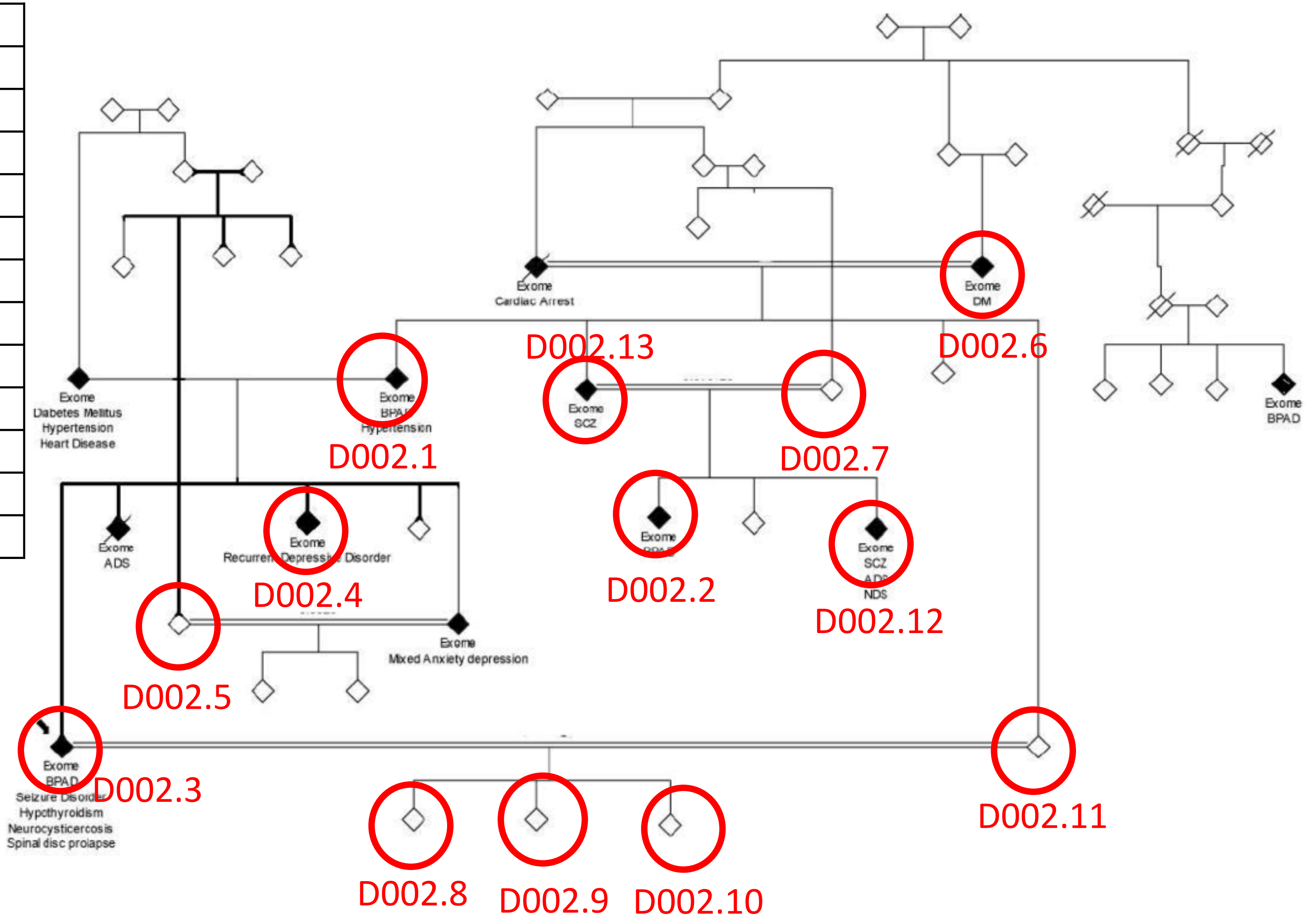

D003

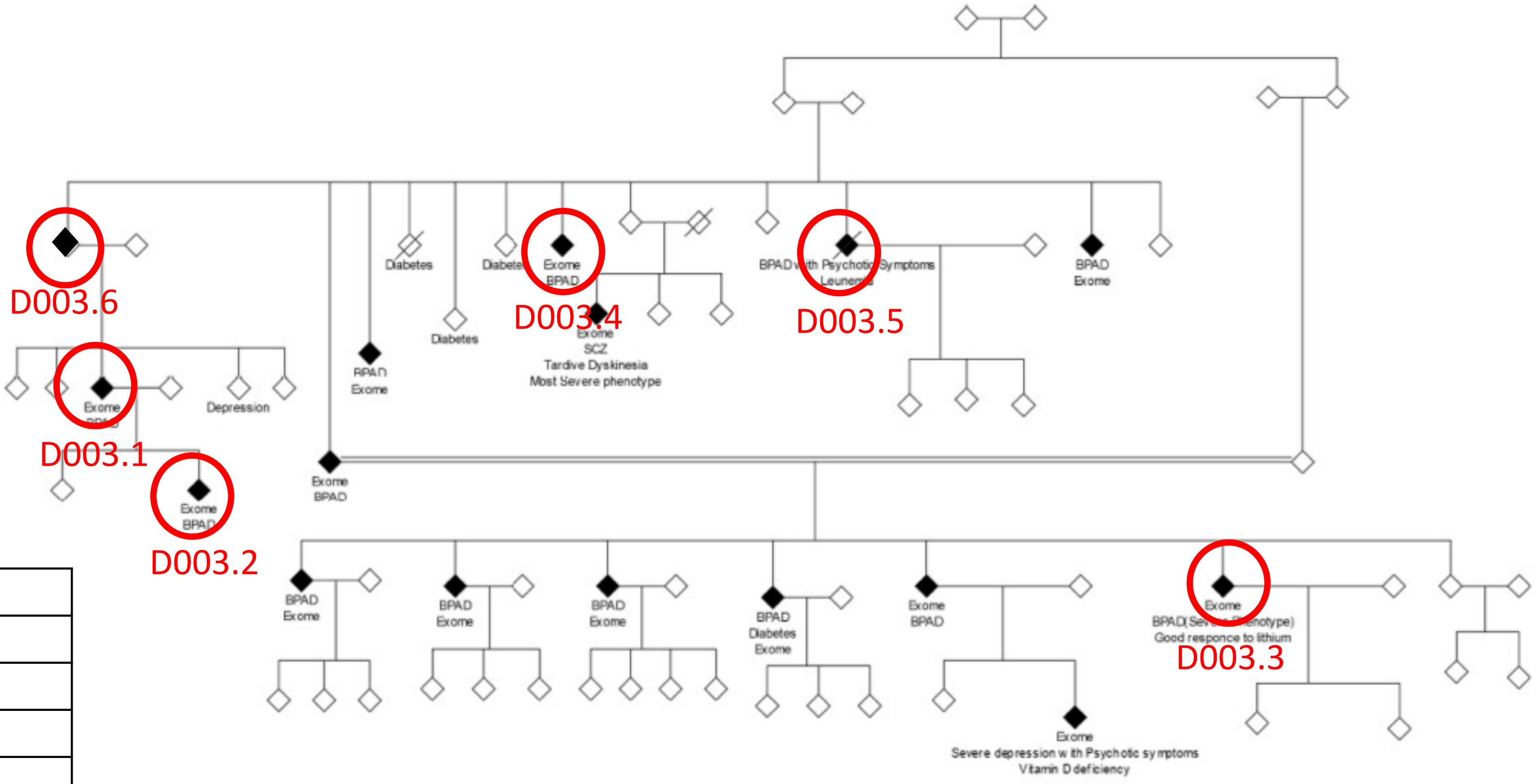

D003.1	BPAD
D003.2	BPAD
D003.3	BPAD
D003.4	BPAD
D003.5	BPAD
D003.6	Schizophrenia

D004

D004.1	BPAD
D004.2	BPAD
D004.3	BPAD
D004.4	BPAD
D004.5	Familial_control
D004.6	Familial_control
D004.7	Familial_control
D004.8	Familial_control
D004.9	Familial_control

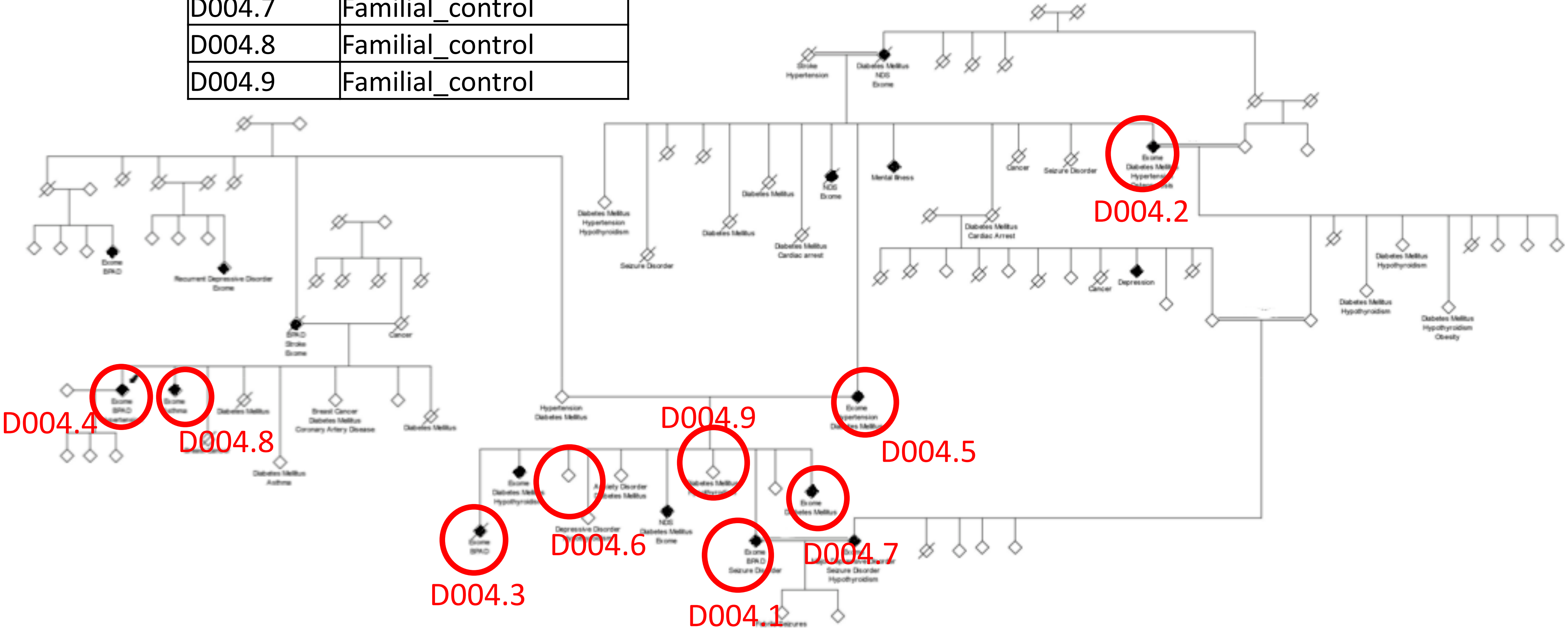

D005

D005.1	BPAD
D005.2	BPAD
D005.3	BPAD
D005.4	BPAD
D005.5	BPAD
D005.6	Familial_control

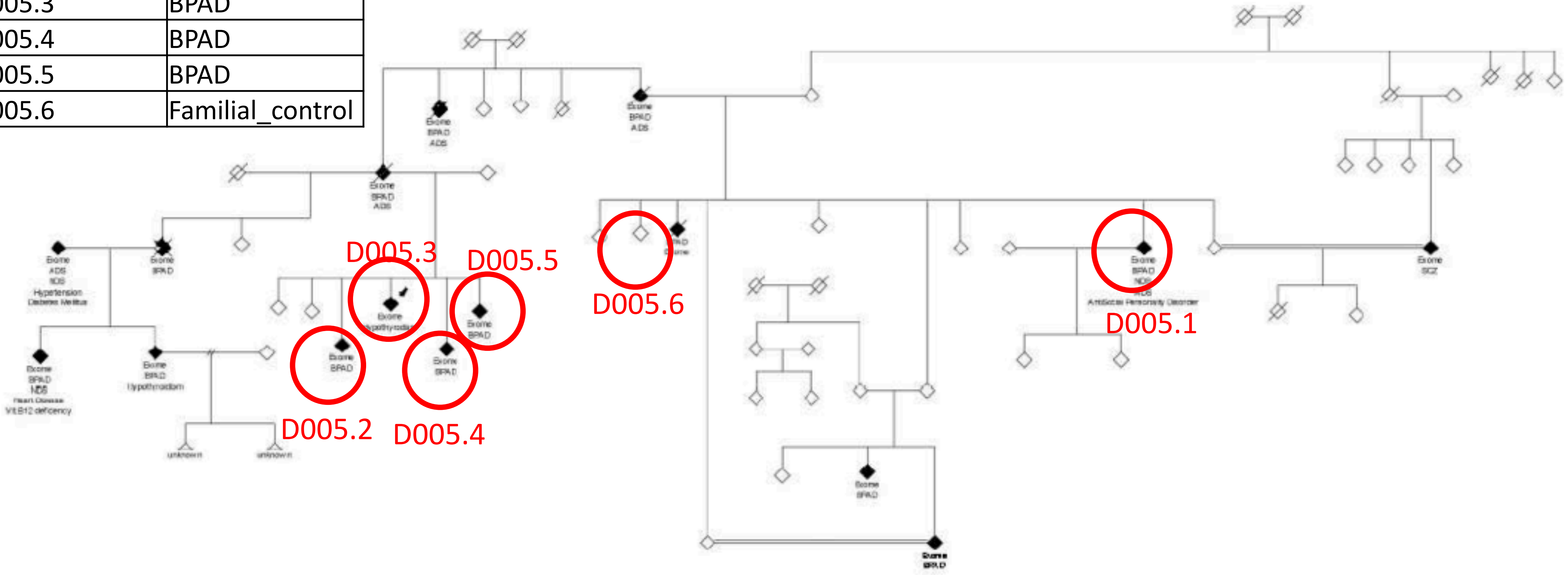

D006

D006.1	BPAD
D006.2	BPAD
D006.3	BPAD
D006.4	BPAD
D006.5	BPAD
D006.6	Familial_control
D006.7	Familial_control

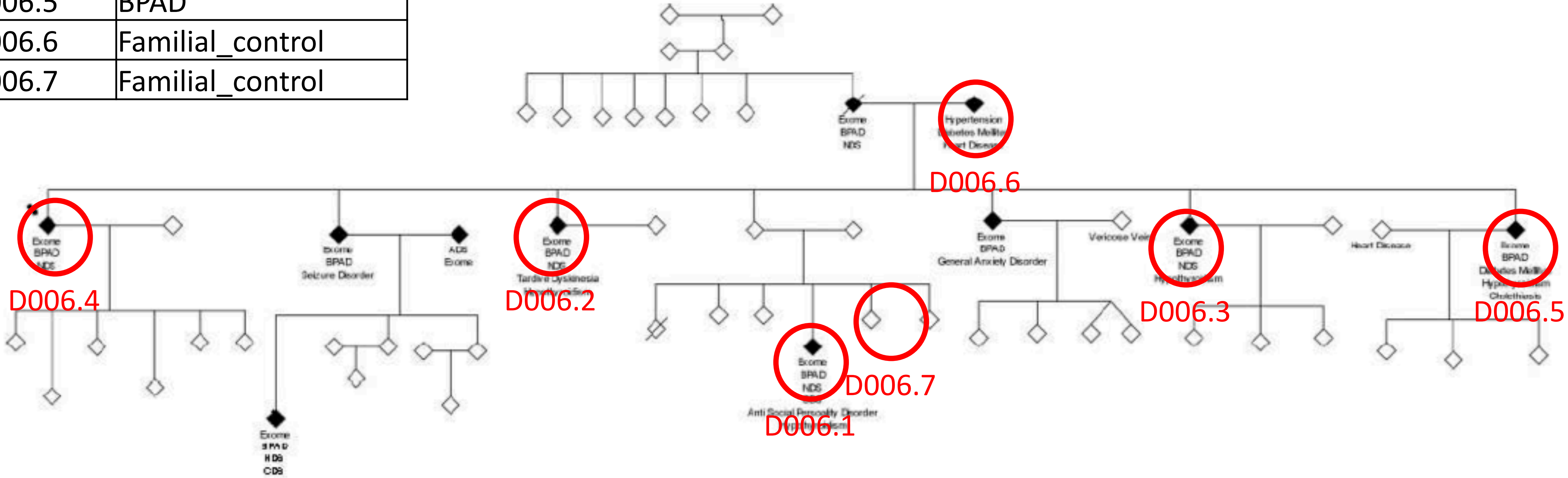

D007

D007.1	BPAD
D007.2	BPAD
D007.3	BPAD
D007.4	BPAD
D007.5	Familial_control
D007.6	Familial_control
D007.7	Schizophrenia

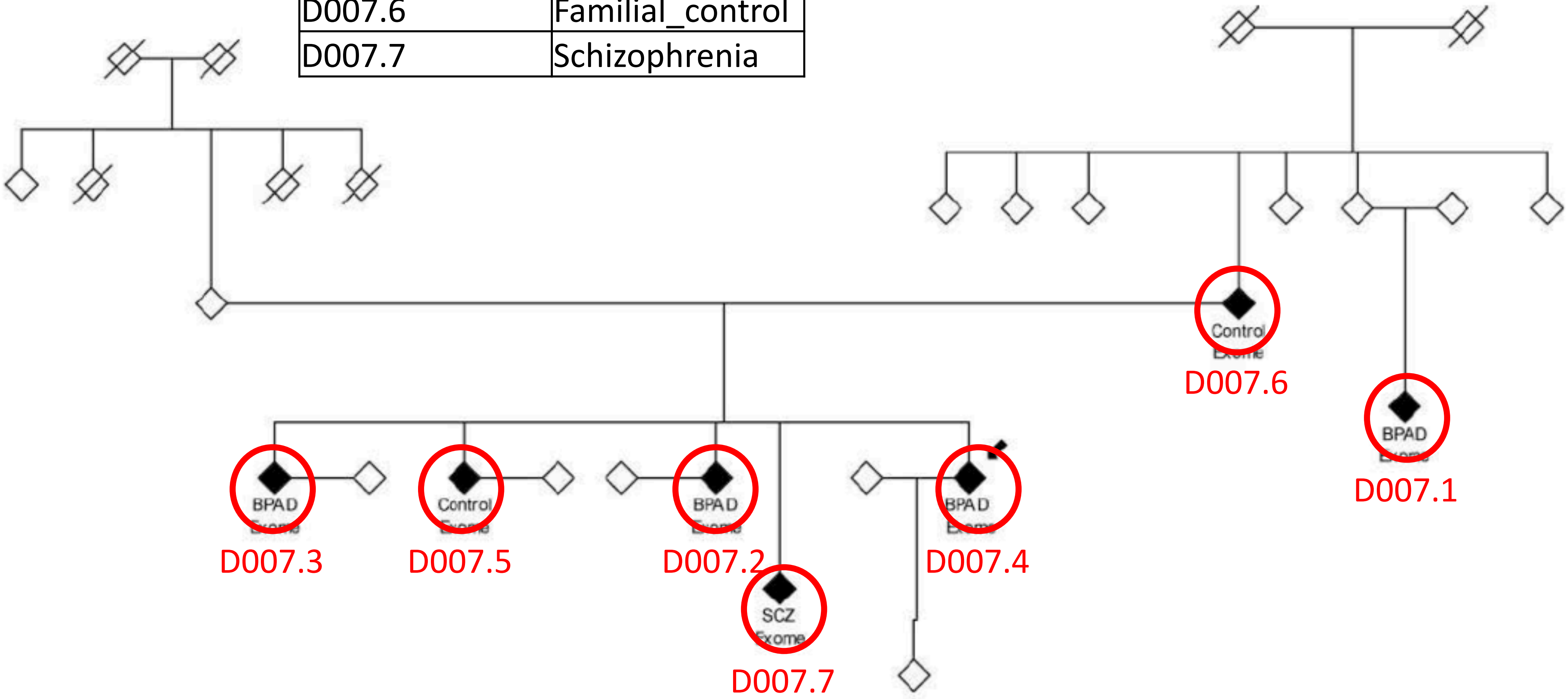

D008

D008.1	BPAD
D008.2	BPAD
D008.3	BPAD
D008.4	BPAD
D008.5	Familial_control

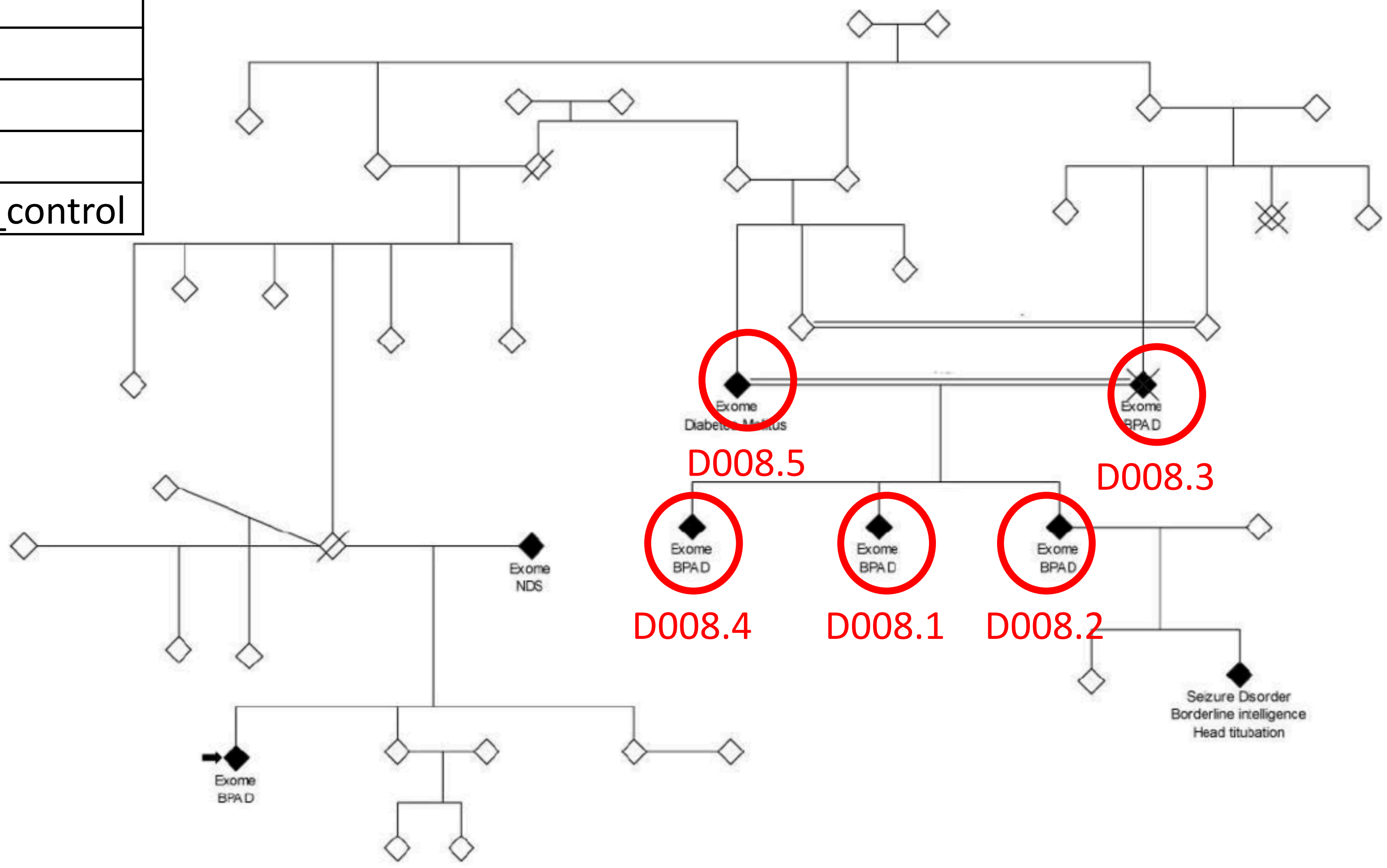

D009

D009.1	Schizophrenia
D009.2	Schizophrenia
D009.3	Schizophrenia

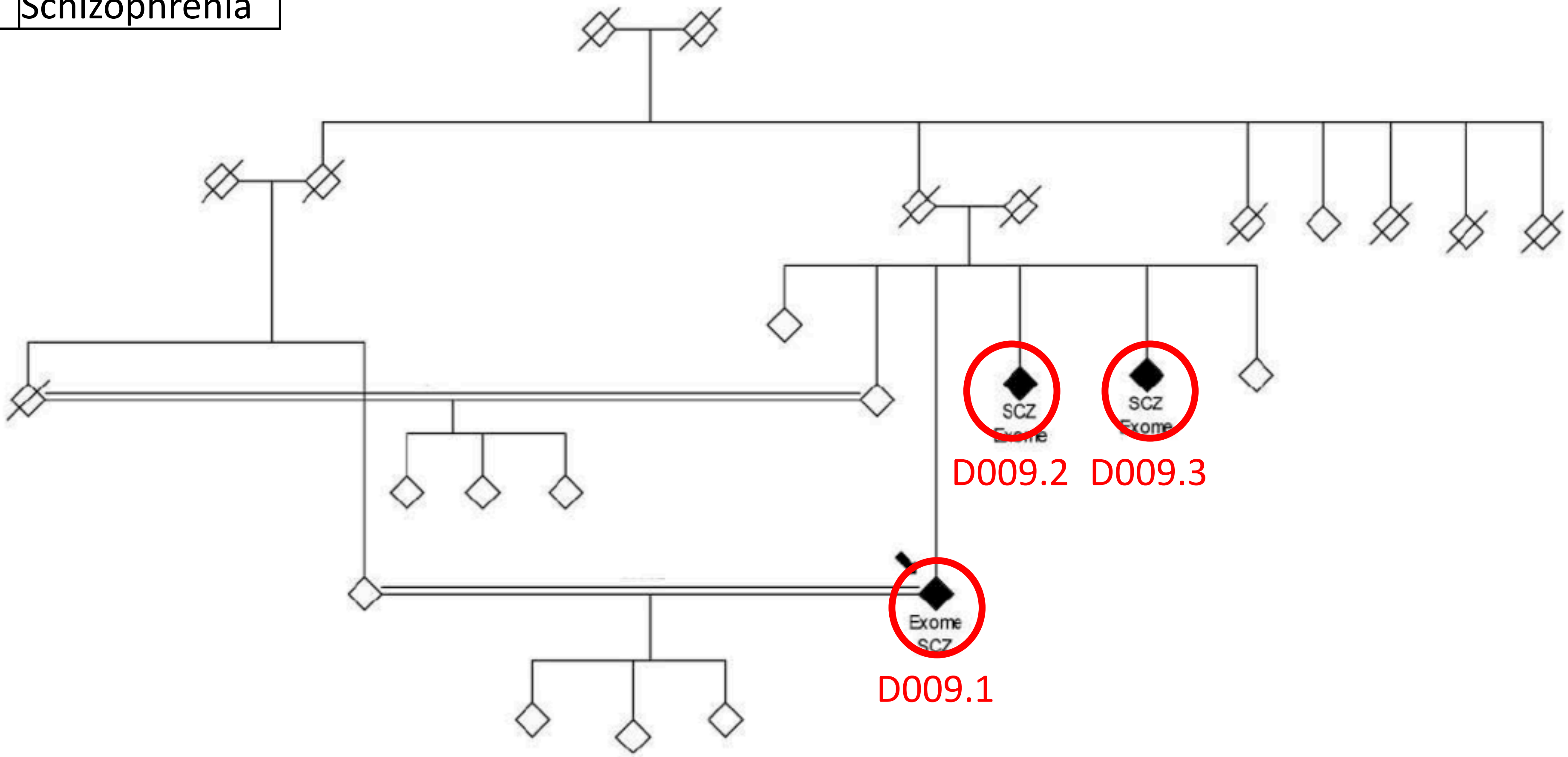

D010

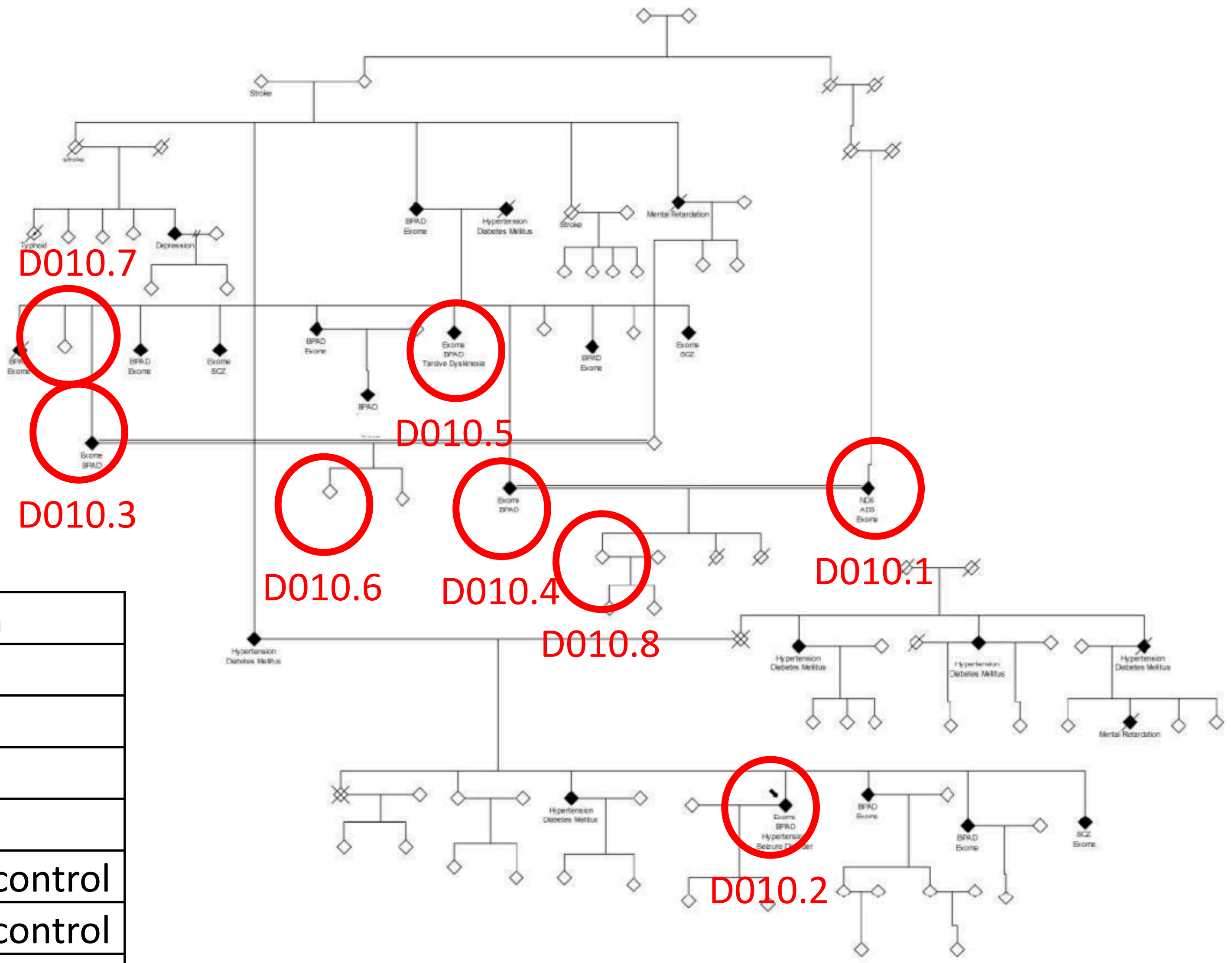

D010.1	Addiction
D010.2	BPAD
D010.3	BPAD
D010.4	BPAD
D010.5	BPAD
D010.6	Familial_control
D010.7	Familial_control
D010.8	Familial_control

D011

D011.1	Schizophrenia
D011.2	Schizophrenia
D011.3	Schizophrenia

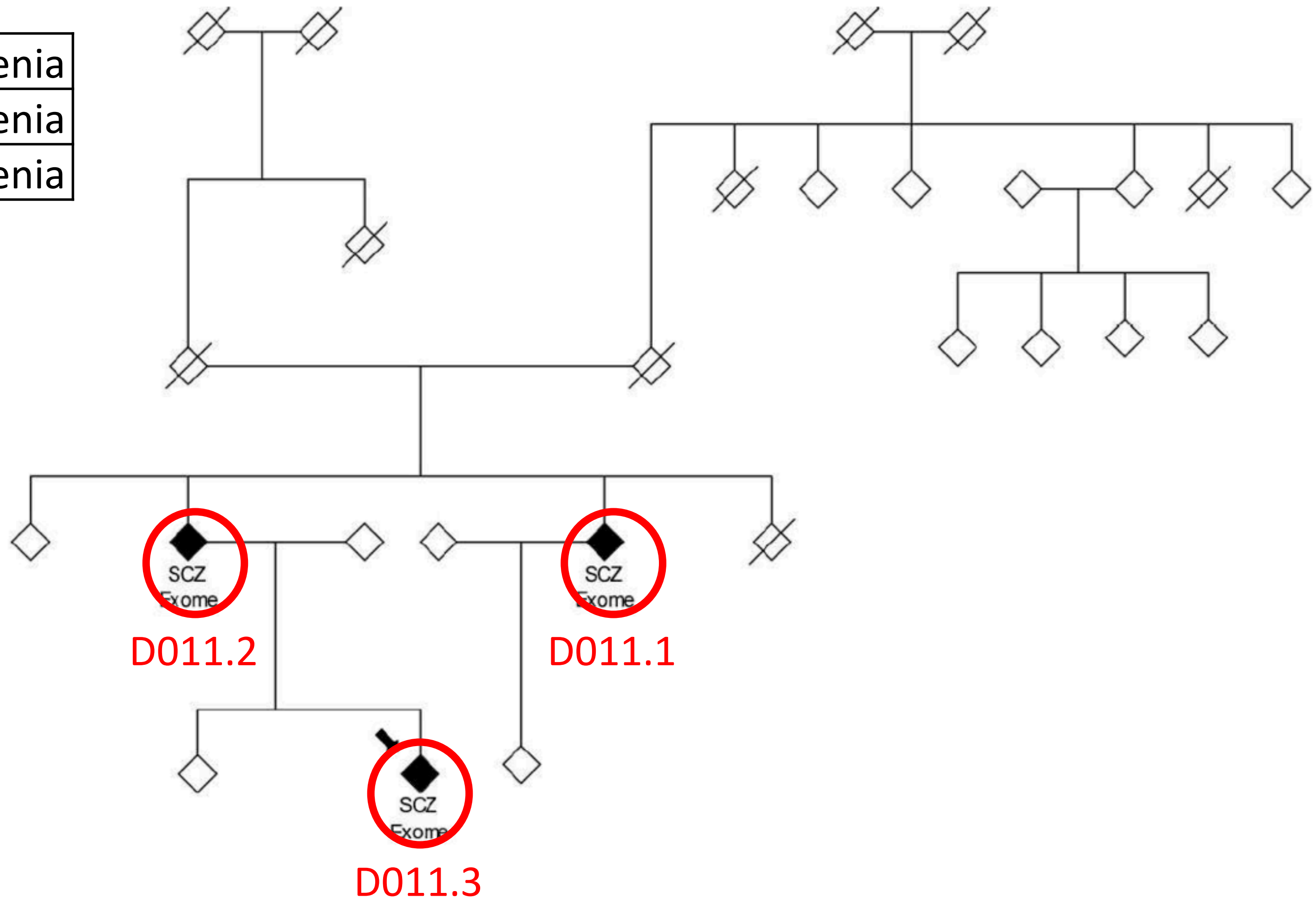

D012

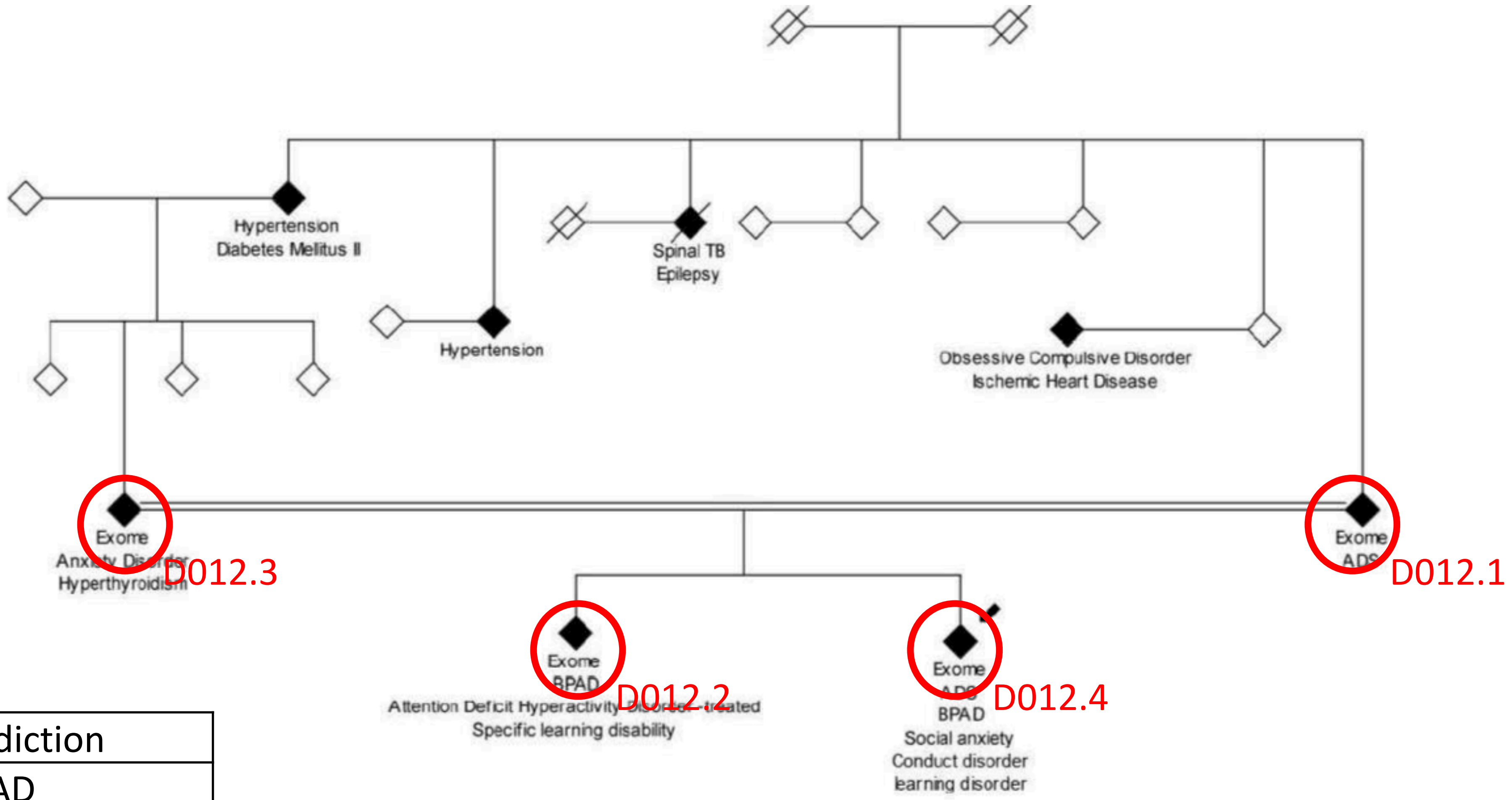

D012.1	Addiction
D012.2	BPAD
D012.3	Familial_control
D012.4	Schizophrenia

D013

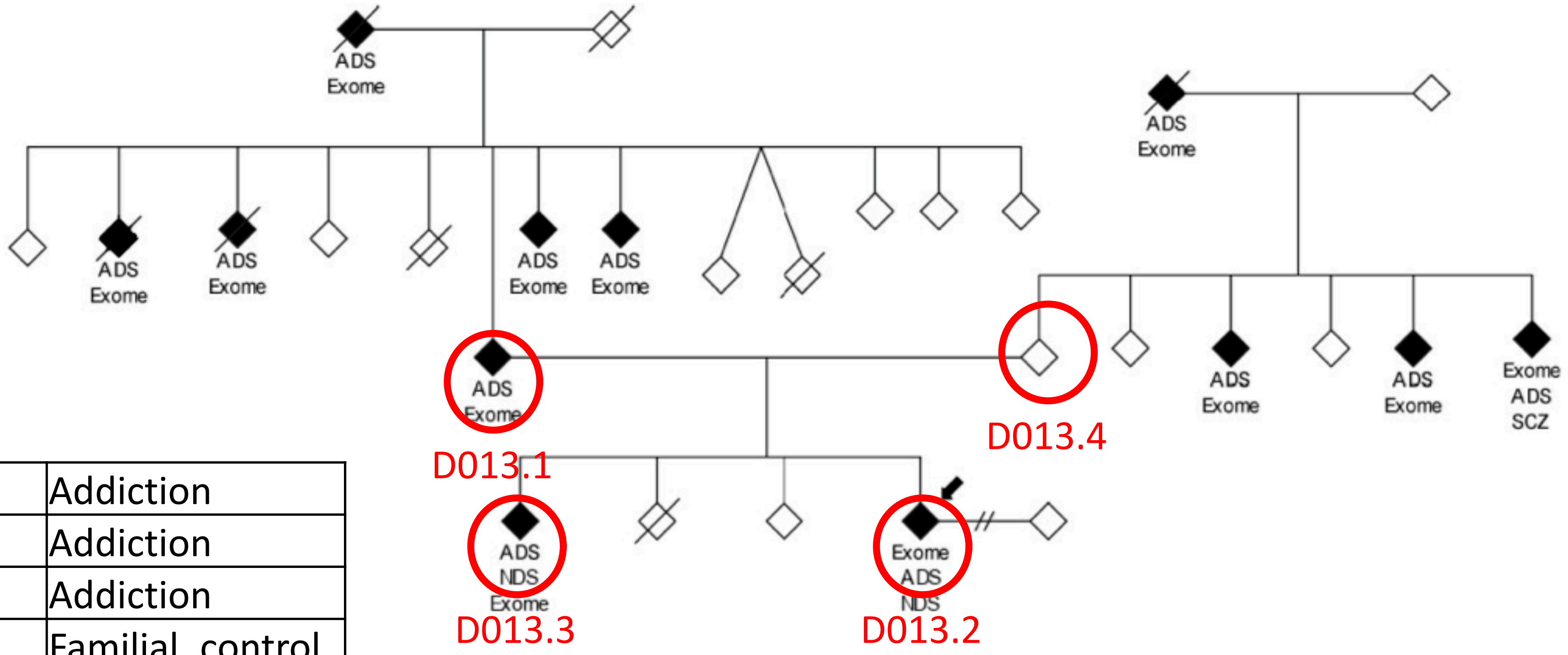

D013.1	Addiction
D013.2	Addiction
D013.3	Addiction
D013.4	Familial_control

D014

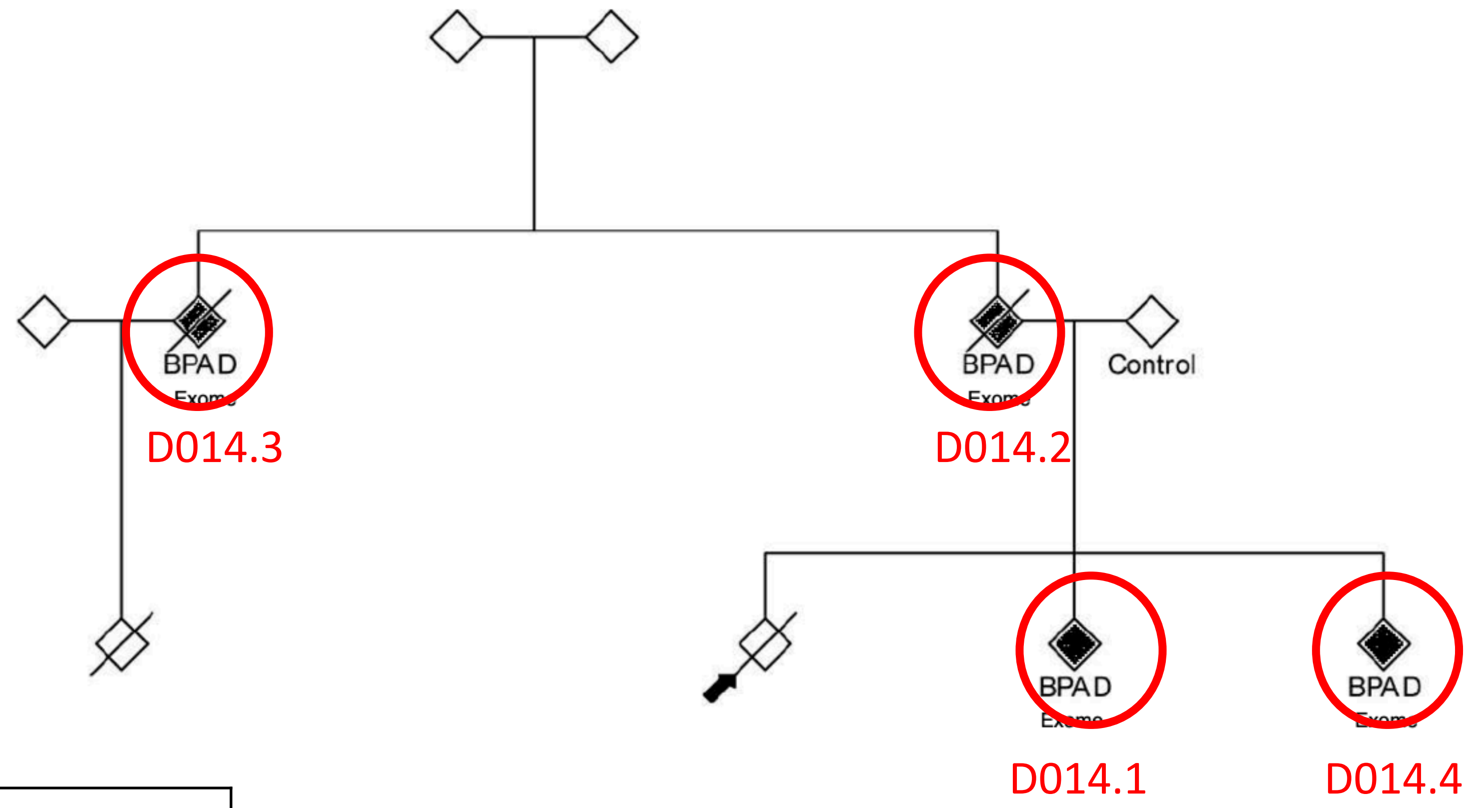

D014.1	BPAD
D014.2	BPAD+Dementia
D014.3	Schizophrenia
D014.4	Schizophrenia

D015

D015.1	Familial_control
D015.2	Schizophrenia
D015.3	Schizophrenia
D015.4	Schizophrenia

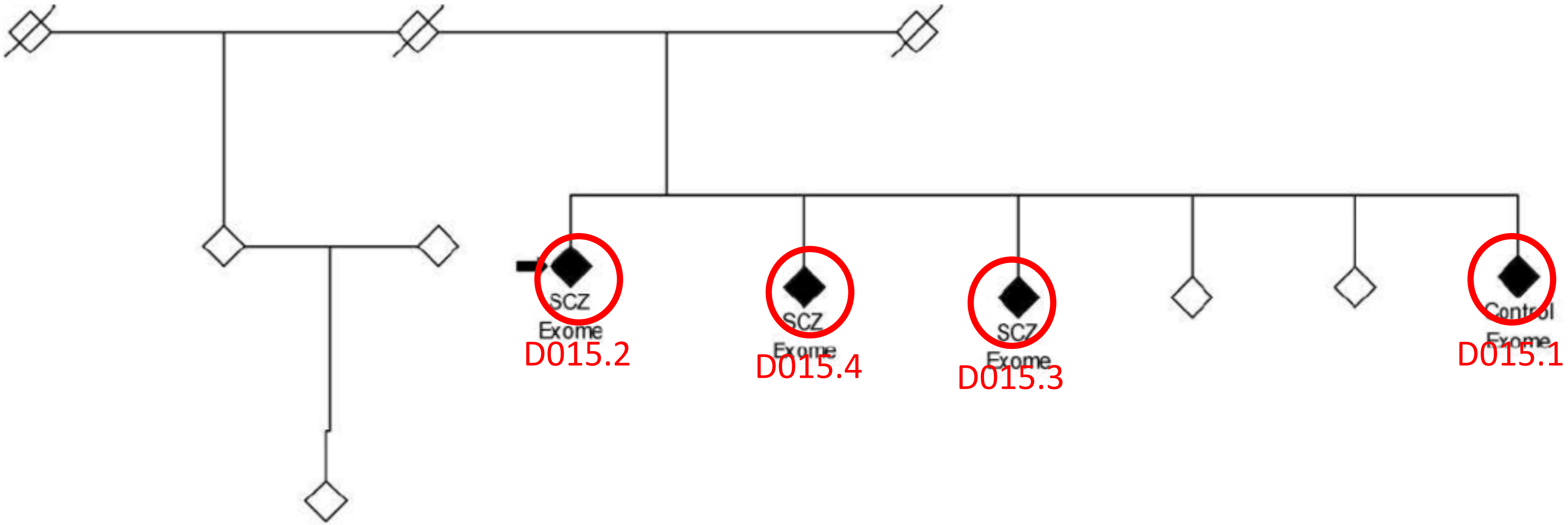

D016

D016.1	BPAD
D016.2	Familial_control
D016.3	Familial_control
D016.4	Schizophrenia
D016.5	Schizophrenia
D016.6	Schizophrenia
D016.7	Schizophrenia
D016.8	Schizophrenia

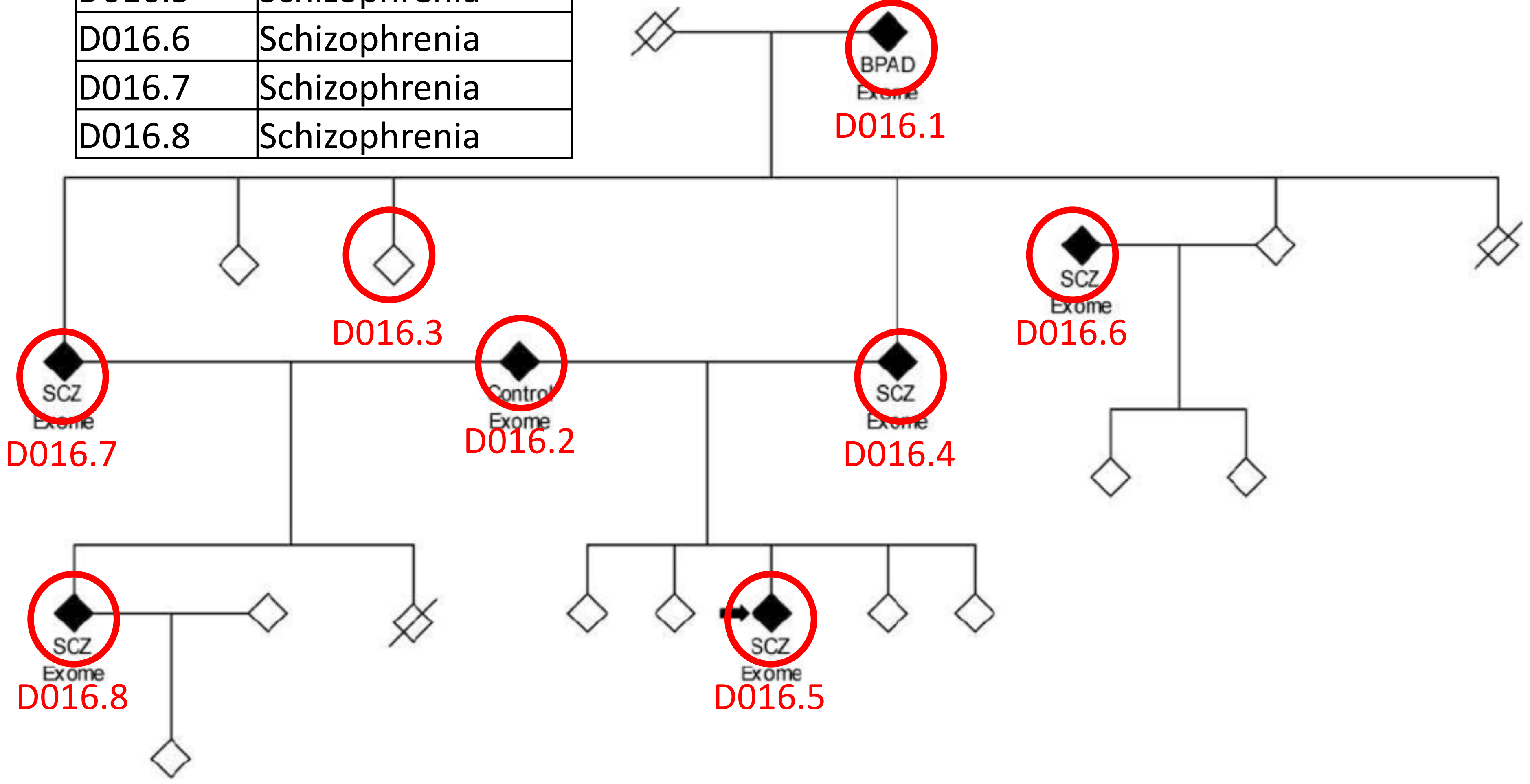

Supplement: Supplementary file 2 — Supplementary Information 2. [file 41598_2022_25664_MOESM2_ESM.pdf]
